# Short communication—Lessons learnt during the implementation of Unity‐aligned SARS‐CoV‐2 seroprevalence studies in Africa

**DOI:** 10.1111/irv.13170

**Published:** 2023-08-23

**Authors:** Elise Farley, Joseph Okeibunor, Thierno Balde, Irene Owusu Donkor, Jackie Kleynhans, Joseph Francis Wamala, Nongodo Firmin Kaboré, Saidou Balam, Dick Chamla, Fiona Braka, Lorenzo Subissi, Belinda Herring, Mairead G. Whelan, Isabel Bergeri, Hannah C. Lewis

**Affiliations:** ^1^ World Health Organization, Africa Regional Office Brazzaville Republic of the Congo; ^2^ Epidemiology Department, Noguchi Memorial Institute for Medical Research University of Ghana Accra Ghana; ^3^ Centre for Respiratory Diseases and Meningitis National Institute for Communicable Diseases of the National Health Laboratory Service Johannesburg South Africa; ^4^ School of Public Health, Faculty of Health Sciences University of the Witwatersrand Johannesburg South Africa; ^5^ World Health Organization Juba South Sudan; ^6^ Centre MURAZ, Institut National de Santé Publique Bobo‐Dioulasso Burkina Faso; ^7^ University Clinical Research Center, Faculty of Medicine and Odonto‐Stomatology University of Sciences, Techniques and Technologies of Bamako Bamako Mali; ^8^ World Health Organization, Head Quarters Geneva Switzerland; ^9^ SeroTracker, Centre for Health Informatics, Cumming School of Medicine University of Calgary Calgary Canada

**Keywords:** methodological challenges, SARS‐CoV‐2, sero‐epidemiological investigations, Unity Studies

## Abstract

The WHO Unity Studies initiative engaged low‐ and middle‐income countries in the implementation of standardised SARS‐CoV‐2 sero‐epidemiological investigation protocols and timely sharing of comparable results for evidence‐based action. To gain a deeper understanding of the methodological challenges faced when conducting seroprevalence studies in the African region, we conducted unstructured interviews with key study teams in five countries. We discuss the challenges identified: participant recruitment and retention, sampling, sample and data management, data analysis and presentation. Potential solutions to aid future implementation include preparedness actions such as the development of new tools, robust planning and practice.

## 1 INTRODUCTION

To enable the rapid implementation of early COVID‐19 sero‐epidemiological investigations to guide policy decision‐making,^1^ WHO developed new or adapted existing,^2^ standardised generic protocols branded as the ‘Unity Studies’; a key focus was supporting low‐ and middle‐income countries to implement these studies.^1^ Unity‐aligned studies meet criteria including aspects of study design, study population, sampling, recruitment and using well performing serological tests.^1^ The most frequently implemented studies in the African region were those using the seroprevalence, transmission and vaccine effectiveness protocols.^3^ As of the 24 February 2023, 179 Unity‐aligned SARS‐CoV‐2 seroprevalence studies had been conducted in the WHO African region.^4^

The main methodological challenges reported in a 2022 external evaluation of the Unity Studies were delays caused by protocol finalisation, ethical approval and gaining access to funding and test kits.^5^ Gaps were also reported in human resources in laboratory science, data analytics and communications.^5^

To gain a deeper understanding of the methodological challenges faced when conducting seroprevalence studies in Africa, we conducted unstructured interviews with purposively sampled study teams that had implemented at least one Unity‐aligned seroprevalence study in Africa (Burkina Faso, NFK; Ghana, IOD; Mali, SB; South Africa, JK and South Sudan, JFW). We present four key reoccurring methodological challenges identified during these interviews and potential solutions to aid future implementation.

### 1.1 Recruitment and retention

Seroprevalence studies require the inclusion of a predetermined minimum number of participants to estimate the population prevalence with good precision.^6^ Teams faced challenges with recruitment and retention of participants and therefore, their ability to meet their minimum sample size.

‘People were reluctant to participate because of fear and stigma.’ (Ghana)

‘When initially enrolling participants into the study, we informed them that we would provide them with their test results as soon as they were available. However, there were delays with the delivery of the test kits provided by WHO, as such, we were unable to get the results to participants before the next round of follow‐up visits. People didn't want to continue with the study and give a new sample before they received their results.’ (Burkina Faso)

These examples add weight to the well documented importance of risk communication and community engagement (RCCE) in surveillance and operational research.^7^, ^8^, ^9^ Community engagement should be implemented as a routine activity of enhanced surveillance. Engagement opportunities include identifying people that the community trusts and building relationships with them and involving them in decision‐making, utilising human resources from the community, and importantly, disseminating study findings with all stakeholders.^7^, ^9^, ^10^ It is likely that enhanced RCCE would improve recruitment and retention.

The Malian team reported that one of the main successes of their study was that:

‘We were able to build a young, dynamic, multidisciplinary team capable of implementing future studies, with involvement of stakeholders at all levels including policy makers, community leaders, epidemiologists, immunologists, biologists, data mangers, sociologists and anthropologists.’ (Mali)

Engagement with and continual support of teams such as these is an important way to ensure timely community‐based investigations during future pandemics.

### 1.2 Sampling

In an idealised setting, a perfect sampling frame (study population from which the sample is selected in order to adequately address study objectives and extrapolate conclusions appropriately to the broader target population) would be a list where each person with known characteristics (e.g., age) is listed once.^11^ Often this does not exist in real‐world settings. At the outset of the investigation, the Burkina Faso team did not have enough information about the population they were going to sample as there was no recent census data. As such:

‘We were obliged to conduct a mini census before we could sample. The last population census was conducted in 2006 and the data needed to be updated. After randomly sampling the enumeration areas (the smallest geographical unit in our setting), we visited the households in these areas and gathered information on the sociodemographic characteristics and sizes of the households. In this way we did a small census to find out about our sample to better establish our sampling frame.’ (Burkina Faso)

This process was time and resource intensive and delayed the start of the study.

Sampling issues also affected the South Sudan team:

‘We used satellite imagery to randomly select shelters for inclusion in the study, some of the sampled households could not be located in the community due to poor internet access in some remote locations which affected the use of GPS to locate the shelters, some households were also empty when study teams arrived, hence the need for supplementary sampling.’ (South Sudan)

Before implementation, partners need to check if an ad hoc census is required and feasible based on available resources and sampling techniques need to be adapted for the constraints of each particular setting, such as limited connectivity.

### 1.3 Sample and data management

Sample and data management was noted as a key area that could be improved for future studies.

Teams mentioned that using easy‐to‐collect samples like dried blood spots (DBS) significantly decreases the complexity around sample management. However, prior validation of serological tests using an appropriate panel of paired samples and a sufficient number of samples, to study test accuracy when using DBS samples is required. Validation of both serological tests and using DBS samples was challenging at the start of the SARS‐CoV‐2 pandemic, which meant that the robustness of non‐validated study results was unknown. As such, it is recommended to use well validated serological tests and sample types where possible and that results are adjusted for test performance (see below).

Manual capturing of participant identifiers on collection forms and samples posed a challenge for teams and led to errors, which further resulted in delays while these discrepancies were resolved. A possible solution suggested by several teams was the development of sample collection standard operating procedures (SOPs) including how samples will be linked through unique identifiers to epidemiological data collected such as through the use of barcodes.

Respondents noted that having access to and training on offline electronic data collection tools is necessary.

‘We used web‐based data collection forms on a platform the team was familiar with as we didn't have time to learn a new software. We had several challenges with connectivity due to limited cell phone network connections in our sampling areas. A better tool would have been a reliable, user‐friendly application that you can capture data with offline.’ (South Africa)

To overcome connectivity issues, some teams used offline mobile data collection tools. They suggested that trainings on how to set up and use these tools should be done in the preparation phase, so that when studies need to be implemented, the tools are familiar to study teams and ready for deployment.

### 1.4 Data analysis and presentation

Teams reported needing assistance with data analysis. The WHO Unity team collaborated with SeroTracker^4^ to provide online workshops and tailored support by producing code and analysis instruction across several analysis tools. Post hoc adjustment support was concentrated on population and clustering adjustment to control for selection bias in participant sampling, and test adjustment to account for potential biases in estimates introduced by serological assay performance (sensitivity and specificity values).

One of the main aims of the Unity Studies is to provide robust evidence for rapid policy decision‐making.^1^ To be effective in this aim, findings need to be presented in a clear and concise manner.^12^ Teams requested assistance with data visualisation and advice on how to best present findings to policy makers.

### 1.5 Tools to address challenges

**FIGURE 1:**
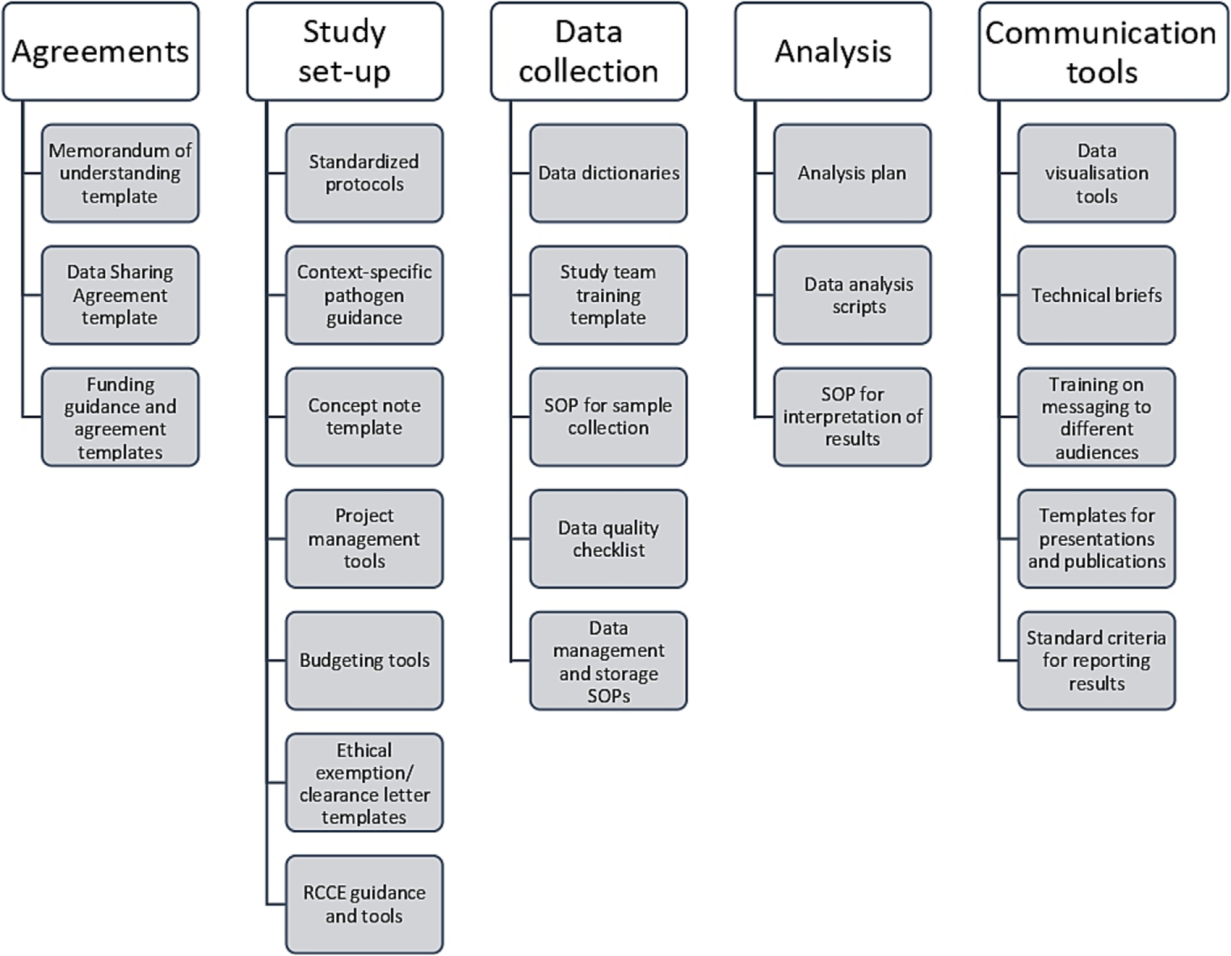
Tools to develop for use in future sero‐epidemiological study and investigation implementation.

Tools (some similar to those identified during the Unity Studies evaluation^5^) to aid future study implementation were identified during the interviews (Figure 1). These included RCCE guidance and tools, sample collection SOPs and access and training in a customizable electronic offline data collection tool. For data management and analysis, teams asked to have access to software in which they are familiar with, a draft analysis plan and data visualisation tools. In addition to updating study protocols for respiratory pathogens of pandemic potential, the Unity Studies initiative intends to generate and distribute such tools with input from partners. To aid the implementation of these tools, trainings and visits between study sites should be encouraged. Implementing study sites have unique public health challenges; as such, the tools created for global application will need to be adapted to fit the context of each study population.

## 2 CONCLUSION

Conducting enhanced surveillance and operational research on a novel pathogen at the start of a pandemic is challenging yet important. The SARS‐CoV‐2 Unity Studies conducted in the African region have proven that these studies are possible to implement at short notice in a robust manner including in low resource settings, and that the resulting information garnered from them is crucial for shaping policy and practice.^5^, ^13^

Investigators should consider the lessons learnt during the SARS‐CoV‐2 pandemic for future surveillance efforts and operational research. Several of the methodological challenges faced can be overcome through preparedness actions such as the development of new tools, robust planning and practice. Countries need to develop resilient well‐coordinated and fit‐for‐purpose surveillance approaches that address priority surveillance objectives not met by current systems for respiratory viruses of epidemic and pandemic potential.^14^ These approaches must be able to be sustained or adapted during an event to ensure an effective response.^14^

## AUTHOR CONTRIBUTIONS

**Elise Farley**: Conceptualization (equal); investigation (equal); writing—original draft (equal); writing—review and editing (equal). **Joseph Okeibunor**: Funding acquisition (equal); writing—review and editing (equal). **Thierno Balde**: writing—review and editing (equal). **Irene Owusu Donkor**: Investigation (equal); writing—review and editing (equal). **Jackie Kleynhans**: Investigation (equal); writing—review and editing (equal). **Joseph Francis Wamala**: Investigation (equal); writing—review and editing (equal). **Nongodo Firmin Kaboré**: Investigation (equal); writing—review and editing (equal). **Saidou Balam**: Investigation (equal); writing—review and editing (equal). **Dick Chamla**: Writing—review and editing (equal). **Fiona Braka**: Writing—review and editing (equal). **Lorenzo Subissi**: Investigation (equal); writing—review and editing (equal). **Belinda Herring**: Writing—review and editing (equal). **Mairead Whelan**: Investigation (equal); writing—review and editing (equal). **Isabel Bergeri**: Funding acquisition (equal); writing—review and editing (equal). **Hannah C. Lewis**: Conceptualization (equal); supervision (lead); investigation (equal); writing—original draft (equal); writing—review and editing (equal).

## CONFLICT OF INTEREST STATEMENT

The authors declare no conflict of interest.

### PEER REVIEW

The peer review history for this article is available at https://www.webofscience.com/api/gateway/wos/peer-review/10.1111/irv.13170.

## ETHICS STATEMENT

This short communication was exempt from ethical approval.

## Data Availability

The dataset supporting the conclusions of this article is available upon request. Requests for access to data should be made to the corresponding author.
